# Upstream development of *Escherichia coli* fermentation process with *PhoA* promoter using design of experiments (DoE)

**DOI:** 10.1007/s10295-020-02302-7

**Published:** 2020-08-25

**Authors:** Frank K. Agbogbo, Phil Ramsey, Renija George, Jobin Joy, Shikha Srivastava, Mian Huang, Jesse McCool

**Affiliations:** 1 Cytovance Biologics, 800 Research Parkway, Suite 200, Oklahoma City, OK 73104 USA; 2 Predictum Inc., Austin, TX USA; 3 University of New Hampshire, Durham, NH USA; 4 BioMarin Pharmaceutical Inc., 770 Lindaro Street, San Rafael, CA 94901 USA

**Keywords:** *E. coli*, Fermentation, DoE, *PhoA*, Model

## Abstract

In this work, a fed-batch fermentation development was performed with recombinant *E. coli* carrying the *PhoA* promoter system. The phosphate concentrations tested for this *PhoA* strain, 2.79 mM to 86.4 mM, were beyond the concentrations previously evaluated for cell growth and product titer. The results from the scouting work was used for design of experiments (DoE) where a range of phosphate levels from 27.1 mM to 86.4 mM was simultaneously evaluated with temperature, pH and DO set points. Definitive screening was used to evaluate these parameters simultaneously and the results indicate that fermentation temperature and phosphate content are the major contributors of product titer. The other factors tested such as pH had a minimal effect and DO had no impact on product titer.

**Electronic supplementary material:**

The online version of this article (10.1007/s10295-020-02302-7) contains supplementary material, which is available to authorized users.

## Introduction

For sponsors of Investigational New Drug Applications (IND) concerning cell-derived biological products, there is the need to develop process knowledge. The scientific understanding of the design space and process will make the scale-up and technology transfer a smooth and effective process. More recent guidelines from the Food and Drug administration emphasizes product and process understanding as well as process control, based on sound science and quality risk management [4, 9, 10]. Development of a robust, well-characterized platform drug substance (DS) upstream process (i.e., high-cell-density fermentation) can avoid costly process development activities in the future. Given that the production in fermenters is a key upstream unit operation in pharmaceutical development, a good understanding of fermentation parameters and their impacts on cell growth [33] and final product yield is critical in defining the process.

*E. coli* is a popular production host for commercializing biological products. Historically, promoters that are inducible by the allolactose analogue, Isopropyl β-D-1-thiogalactopyranoside (IPTG) have been heavily used in the biologics industry. There are some industry-recognized disadvantages associated with IPTG-based induction processes that are related to stress on the cells [14, 15] and cost of goods [3, 7]. Many alternative promoter systems are also available to produce therapeutic proteins at large scale in *E. coli*. Promoter systems such as the arabinose-inducible promoter (pBAD), the heat-inducible phage Lamba promoters (pL and pR), the salt-inducible promoter (*proU*), the cumate-inducible T5 promoter-based system [3], and the cold-inducible *cspA* promoter-based system [30] are potentially useful in the biologics industry [18, 20, 25]. Similar limitations, however, are inherent to those promoter systems due to the need for complex process control and higher raw material costs. In contrast, promoters that allow for auto-induction are more attractive to biologics manufacturers because of the simplicity of the process and the reduced raw materials costs.

In the auto-inducible *phoA* promoter system [22, 28, 29, 31, 32], as the cell culture grows, the phosphate concentration in the culture media drops to a threshold that triggers promoter activity and expression of the target recombinant protein. Compared to other systems, *phoA* system avoids the use of expensive and/or toxic chemical inducers. Furthermore, it reduces the risk of contamination of culture due to in-process addition of chemical inducers. The mechanism by which bacteria respond to phosphate starvation, known as the Pho regulon, is one of the most sensible and efficient regulatory systems in bacteria [16, 22, 28]. Previous studies on fed-batch fermentation using *PhoA* expression system used low concentrations of phosphate and, therefore, achieved low concentrations of biomass and product titers [8, 27, 31]. The goal of this study is to evaluate phosphate concentrations beyond levels that were previously tested in *PhoA* processes and look at the impact of process parameters on cell growth and product titer.

Design of experiments (DoE) comprises a large number of methods used for the efficient, systematic study of physical processes and systems. In performing DoE, multiple experimental factors each at two or more levels are simultaneously manipulated by the experimenters to understand causal relationships occurring in the system or process in study; we refer to this strategy as factorial experimentation. The experiment is conducted in such a manner that all sources of potential variation impacting the response of interest are controlled giving the experimenters information on which of the factors and their effects explain the majority of the observed variation in the response. It is necessary to simultaneously manipulate the experimental factors to understand and estimate the interactions among the factors. In physical systems, the inputs or experimental factors do not act upon the response in an individual manner, but rather they act in concert. The interactions in biological and chemical systems are often the dominant effects on the response. How each factor impacts a response individually is typically far different than how the factors work in combination, i.e., interact. DoE is a very large topic and a full discussion is beyond our scope; for more in depth coverage, see [17].

The causal relationships determined from DoE are expressed in the form of statistical or empirical models, which are typically linear models. The terms or, we often say effects, in an empirical model are typically first-order effects (main effects), interaction effects (cross product terms), and polynomial terms (often quadratic effects). A specific DoE structure is required to estimate the set of desired effects; therefore, the choice of a specific design requires the experimenters to consider the potential effects of the system being studied. There are a very large number of experimental designs to choose from that support various types of empirical models, for a discussion on design structures and empirical models see [17].

Definitive screening designs (DSD) were developed by Jones and Nachtscheim [11, 12] and have become popular in biopharma due to the relatively small number of trials required to estimate experimental effects. The DSD uses three levels for each factor, which allows for the estimation of possible nonlinear effects, while the total number of runs required for K factors is 2 K + 1 if K is even or 2 K + 3 if K is odd; as a general rule, the design for K = 6 is used if K < 6. Usually a small number of additional factorial trials or center point trials are added to the base design to allow for a better estimate of experimental error, check for lack of fit, and model building. Although the DSD is thought of as a screening design they are also very efficient response surface designs since they can be used to estimate two-way interaction terms and quadratic terms. In the current experimental work, DSDs were employed.

The upstream process development was performed for cell growth and product titer in a fed-batch high-cell-density fermentation of *E. coli* where the recombinant protein was being expressed from a plasmid derived copy of the gene under control of the *phoA* promoter [2, 13, 23, 28]. A scouting study was performed to evaluate phosphate concentration levels of 2.79 mM to 86.4 mM, which is beyond concentration levels previously evaluated in the literature (0.43 mM to 18 mM) [8, 27]. The results from the scouting work was used to guide the design of experiments (DoE) work where multiple parameters, phosphate content, temperature, pH and DO, were simultaneously evaluated. Definite screening design (DSD) was employed to evaluate these parameters simultaneously and determine the impact of each of the parameters on product titer.

## Materials and methods

Shake flask inoculum preparation for fermentation: A vial of a research cell bank of *Escherichia coli* strain CBM048 (a *phoA* promoter expression strain based on *E. coli* K-12 W3110 developed using the Keystone Expression System by Cytovance Biologics in Oklahoma City, OK) was used to inoculate 400 mL of BBL™ Select APS™ LB Broth (Becton–Dickinson) supplemented with 12.5 µg tetracycline/mL in a 2-L baffled shake flask. The BBL™ Select APS™ LB Broth was made by following the manufacturer’s instruction. The inoculum was added to the flask at inoculum/media (1/1000, v/v) dilution. Each shake flask involved in this study was put in an incubator with 1-in. throw for 16–18 h at 30 °C shaking at 250 rpm. The OD_600_ was measured after 16 h of incubation. Once the OD_600_ was in the range 5.0–7.0, the culture was transferred to the 5-L fermenter.

Fermentation: Fed-batch fermentation was performed in 5-L New Brunswick BioFlo 115 (Eppendorf) with different phosphate concentrations in the batch medium: 2.79 mM, 27.1 mM, 40.1 mM, 58.9 mM, and 86.4 mM. The DoE fermentations were performed in 5-L Sartorius Biostat A^+^ Fermenters. The batch medium consisted of 29.9 g soytone hydrolysate/L, 14.8 g yeast extract/L, 11.6 mL of 50% glycerol/L, and 0.78 g sodium citrate dihydrate/L. The volume of the batch was 2.35 L (beginning of fermentation) in each 5-L fermenter and the target final volume of the fermentation was 4.5 L (end of fermentation). The batch medium was autoclaved at 121 °C for 45 min. After the batch medium was cooled to room temperature, it was supplemented with 10 mL of 20% antifoam 204, 48.4 mL of trace element solution/L, 2.40 mL of 12.5 µg/mL tetracycline solution/L, 45 mL of 1 M MgSO_4_ and 250 mL of mineral salt solution supplement. The mineral salt solution composition used for the scouting study [24] is presented in the Supplementary Material. A similar mineral salt composition was used for the design of experiments (DoE). Foam production was controlled during the fermentation by the addition of a 20% Antifoam 204. The pH was controlled with 30% (v/v) ammonium hydroxide and 10% (v/v) sulfuric acid. The fermentations were performed as a fed-batch process at 30 ± 0.5 °C, pH of 6.8 ± 0.1, airflow of 4.5 Lpm (~ 1 vvm), and dissolved oxygen (DO) controlled at 35% ± 5% or the required conditions in the DoE table (Table 3). Oxygen supplementation was used for DO control at high cell densities.

After the fermenter was batched and brought to set point conditions, it was inoculated with 1% inoculum culture (v/v) (23.5 mL) based on the starting batch volume. The fermentation was run as a batch fermentation until the carbon source in the medium was depleted. A DO spike occurred when the carbon source was depleted at which point, glucose feed was initiated with the following levels 17% glucose (v/v) (2.79 mM), 27% glucose (v/v) (27.1 mM), 32% glucose (v/v) (40.1 mM), 39% glucose (v/v) (58.9 mM) and 50% glucose (v/v) (86.4 mM). Different glucose concentrations were supplied to match the expected increase in biomass at the different phosphate levels tested. The culture was harvested after 48 h of elapsed fermentation time from the time of inoculation. The volume of feed expected to be delivered during the entire fermentation is 1.8 L. The fermentation broth of 100–200 mL at the end of the fermentation was taken and stored at −20 °C.

### Measurements of cell biomass concentration

The cell biomass concentration of fermentation cultures was estimated by both optical densities at a wavelength of 600 nm (OD_600_) and dry cell weight (DCW). For OD_600_ measurements, in-process samples were appropriately diluted with deionized water to obtain OD_600_ values between 0.1 and 0.9. The reported OD_600_ of the cell culture samples is the product of the measured value and the dilution factor. The OD_600_ measurements were performed by DU^®^730 (Beckman Coulter). For DCW measurements, 10 mL of samples were centrifuged for 5 min at 14000 rpm and washed three times with deionized water. Pellets were resuspended in 1 mL of water transferred to a pre-weighed aluminum dish and dried overnight at 105 °C until no further difference in weight. The mean values of DCWs of three independent measurements and their standard deviations are reported in g/L.

### Measurements of phosphate concentration

Samples of fermentation cultures were taken at selected time points for the analysis of phosphate concentration of the clarified supernatants. Measurements were made using the Cedex Bio analyzer (Roche). The measurements followed the manufacturer’s instruction.

### SDS-PAGE

The broth that was taken during fermentation was thawed and centrifuged at 10,000×*g* for 10 min at 4 °C. The paste collected was then weighed and then mixed with 9x the weight of nanopure water (1:9). The mixture was then homogenized in PANDA (GEA Separations) at 10,000 psi for ~ 2 min. After two passes through the homogenizer, 1 mL of each homogenized sample was then centrifuged at 10,000×*g* for 10 min to remove the supernatant. The pellets were then weighed and suspended in 1 ml of 1X reducing buffer. The 1X reducing buffer comprises 1X NuPAGE^TM^ LDS Sample Buffer (ThermoFisher Scientific), 0.025 M DTT, and nanopure water. 20 µL of the suspension was further diluted with 1X reducing buffer to an appropriate concentration between 0.00625 µg/µL and 0.05 µg/µL. The resulting solution was incubated at 70 °C for 2 min before being loaded onto 12% Criterion™ XT Bis–Tris Protein Gel (Biorad) and separated at 200 V for 50 min. 20 µL of the protein reference standards (Sigma-Aldrich) with a concentration gradient of 0.00625, 0.0125, 0.025, and 0.05 µg/µL was loaded onto the gel as well for estimation of the target protein concentration in the samples. The gel was then stained with Coomassie stain, de-stained and imaged for densitometric analysis of concentration using GeneTools software (Syngene). The average and standard deviation of the titer were calculated from three replicate runs of each sample analyzed.

### Design of experiments (DoE)

The JMP® Pro 15 statistical software was used to generate the design and perform the analyses. For process characterization, optimization of an empirical model called the full quadratic model is utilized [1]. The full quadratic model includes all main effects (first-order terms), all quadratic effects (second order terms), and two-way interaction effects (cross product terms). The quadratic and interaction terms allow the model to accommodate nonlinear and non-additive relationships between the experimental factors and the responses. Equation 1 is an example of a theoretical full quadratic model for K = 3 factors. Experimental designs that estimate the full quadratic model are often referred to as response surface designs.

1 $$Y = \beta_{o} + \beta_{1} X_{1} + \beta_{2} X_{2} + \beta_{3} X_{3} + \beta_{11} X_{1}^{2} + \beta_{22} X_{2}^{2} + \beta_{33} X_{3}^{2} + \beta_{12} X_{1} X_{2} + \beta_{13} X_{1} X_{3} + \beta_{23} X_{2} X_{3} + \varepsilon .$$

The original response surface designs were developed by Box [1] to estimate the entire quadratic model for a set of experimental factors K. The Central Composite Design (CCD), invented by Box, continues to be very popular for process characterization and optimization. Unfortunately for K ≥ 4, these designs become prohibitively large and more efficient alternative designs are required.

## Results and discussion

The product of interest is expected to be expressed following the depletion of phosphate in the batch medium. There five conditions tested in the scouting work had different initial amount of phosphate. The responses were final biomass production measured by dry cell weight and OD_600_, and final product titer measured by SDS-PAGE and scanning densitometry. Table 1 shows the dry cell weight, OD_600_, and product titers obtained for each initial phosphate concentration during scouting work. As the phosphate concentration increased from 2.79 mM to 86.4 mM, the biomass increased as measured by OD_600_ (33 to 234) (Table 1) and dry cell weight (9.43 g/L to 77.5 g/L) (Table 1). Since phosphate is contained in nucleic acids, lipids, proteins, and sugars, and is involved in many biochemical reactions within the cell, an increase in phosphate is expected to result in an increase in biomass production. The optical density reported in this work, 234, is higher than 6.6 [8] and the dry cell weight from this work, 77.5 g/L, is higher than 30 g/L [31] or 13 g/L [27]. These data demonstrate that more cell mass is generated as the phosphate concentration is increased beyond levels that have been previously reported (10.8 − 18.0 mM) [27] and (0.43 − 17.0 mM) [8] for a *PhoA* process.

**Table 1:** OD_600_, dry cell weight, product concentration, quantity of glucose delivered and phosphate concentration after 48 h of fermentation at the different conditions tested in this study. The dry cell weight and product concentration values are mean and standard deviation of three independent analysis of the samples

Initial phosphate concentration (mM)	OD_600_ at harvest^a^	Dry cell weight (g/L)^b^	Product concentration (g/L)^c^	Quantity of glucose feed delivered (g)	Phosphate concentration at harvest (mM)
2.79	33.0	9.43 ± 1.79	0.443 ± 0.200	1695	0
27.1	103	36.0 ± 5.28	2.18 ± 0.494	1963	0
40.1	114	44.9 ± 6.88	0.796 ± 0.419	1725	0
58.9	153	63.8 ± 2.04	1.08 ± 0.292	1741	0
86.4	234	77.5 ± 10.2	3.48 ± 0.633	2001	0

^a^The OD_600_ measurements are within 10% of the measured value

^b^The dry cell measurements are ± 1 std

^c^The titer measurements are ± 1 std

**Fig. 1:**
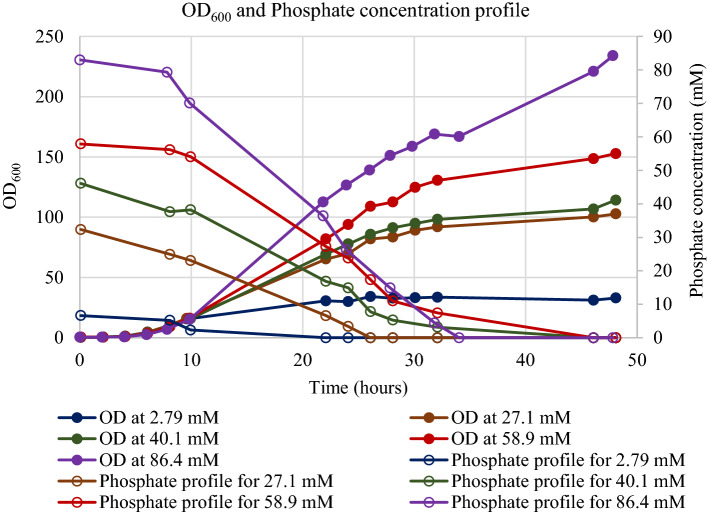
OD_600_ and phosphate concentration values generated from scouting fermentation conditions using different initial amount of phosphate. The concentration of phosphate in the media from the start of the fermentation till the end was measurement using the Cedex BioAnalyzer

**Fig. 2:**
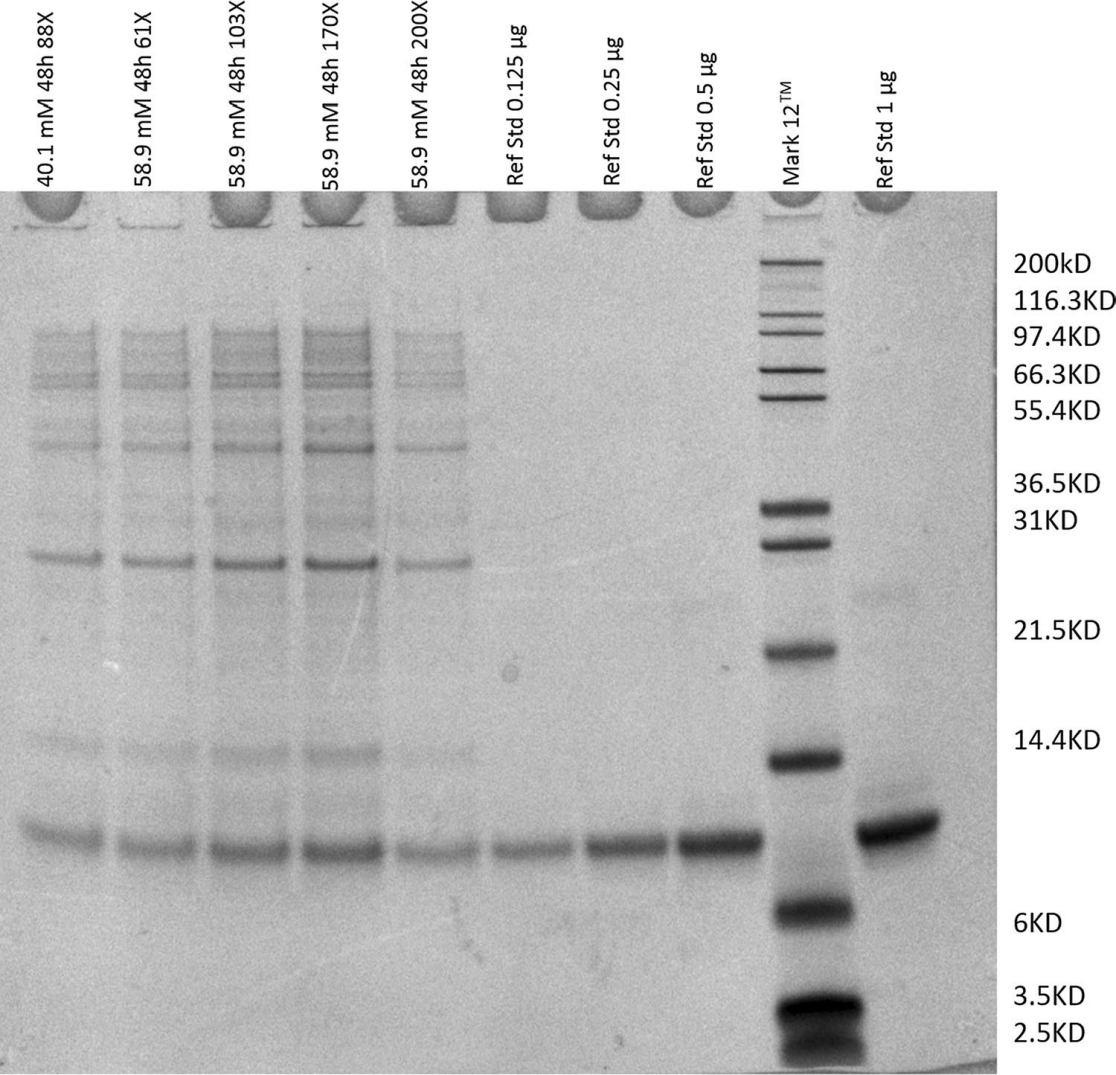
SDS-PAGE gel from harvest samples with 40.1 mM and 58.9 mM initial phosphate concentration, reference standard at different concentrations and a ladder. The dilutions 61X, 103X, 170X and 200X are based on the dilutions of the initial broth up till the point of loading on the gel. The reported titers are the mean and standard deviations calculated from densitometric analysis of concentration using GeneTools software (Syngene)

Multiple time-point samples were tested for phosphate concentration from the start of fermentation till the end of fermentation. From the profiles in Fig. 1, the phosphate concentration was decreasing as the fermentation progressed and phosphate levels at harvest were zero. The pellets at the end of fermentation were analyzed for the presence of products. The insoluble fractions of the cells after homogenization were analyzed and compared with the reference standard (Fig. 2). The titer and mass of glucose delivered during the entire fermentation are shown in Table 1. The data show the product titer increased from 0.443 to 3.48 g/L as the phosphate concentration increased from 2.79 to 86.4 mM.

The lowest concentration of phosphate tested (2.79 mM) showed the lowest titer of 0.443 g/L and the highest phosphate tested showed the highest titer of 3.48 g/L. The acetate concentration was higher at the lowest phosphate level tested (2.79 mM) compared to the other levels of phosphate tested in the fermentation (Supplementary Material). The data showed that the reduction of glucose levels to match the phosphate concentration was not low enough since the cells appeared to have been overfed at 2.79 mM. The titers reported in this work 3.48 g/L for this protein is higher than 0.327 g/L [31] or 0.35 g/L [27]. This work demonstrates that the product titer can be improved as the initial phosphate concentration is increased beyond levels previously reported in the literature.

**Fig. 3:**
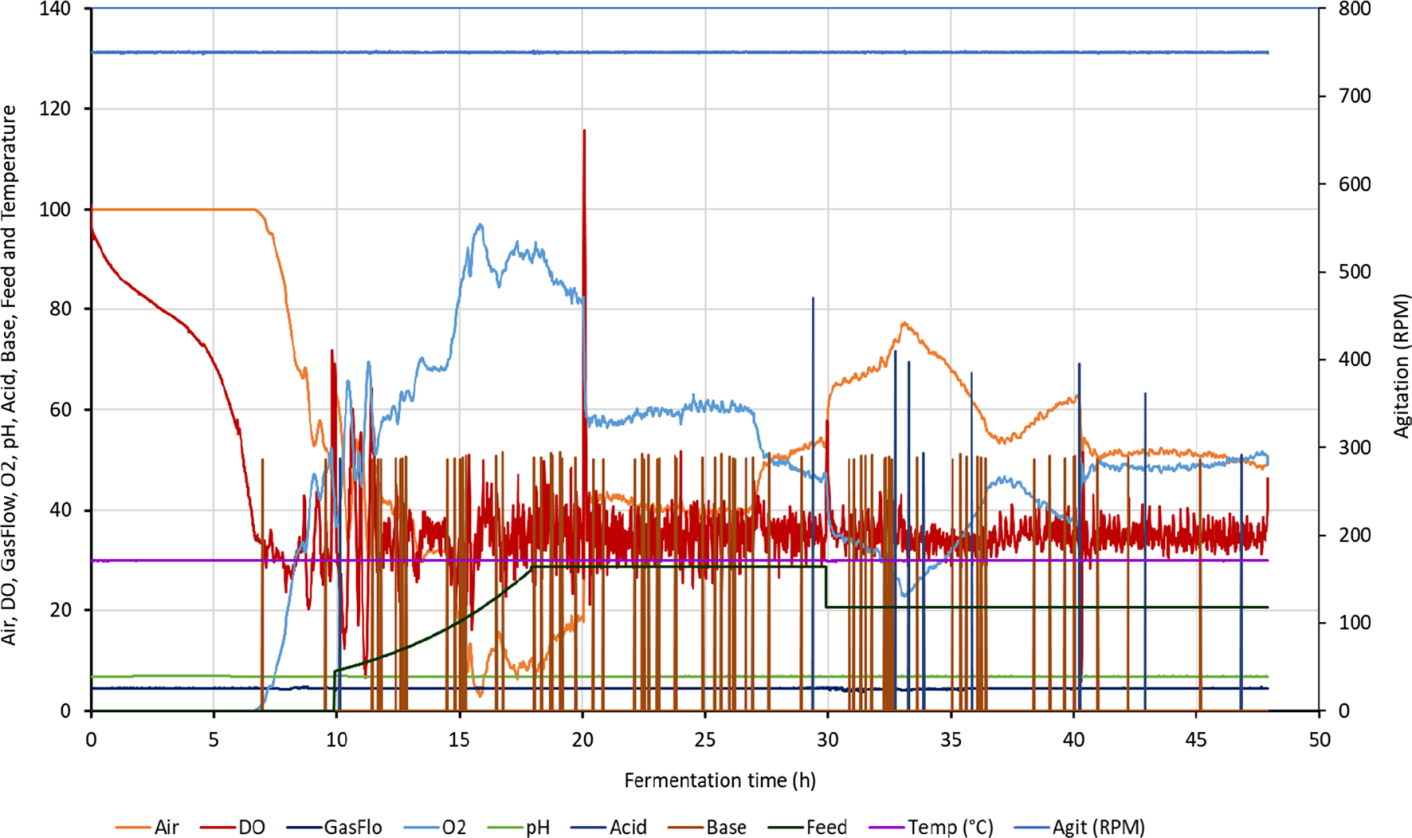
On-line profile for 86.4 mM initial phosphate fermenter for air (%), DO (%), gas flow (L/min), O_2_ (%), pH, acid (% duty cycle), base (% duty cycle), feed (% duty cycle), temperature (°C) and agitation (RPM). The total gas flow was kept at a constant value of 4.5 L/min consisting of a mixture of air and oxygen. The percentage of the total gas flow that is air and oxygen is represented in the graph. The feed profile was initiated after the DO spike with an exponential feed initially and a constant feed

The production fermentation system controlled for temperature, pH, DO, stirrer speed, gas mixing, and substrate. The DO control strategy used in these fermentations is to start at a fixed agitation of 750 rpm and when DO gets to the set-point, the DO is controlled with oxygen supplementation. A sample of the on-line data generated during the fermentation is shown in Fig. 3.

### Design of experiments (DoE)

To characterize and optimize the fermenter performance, a 4-factor DSD with 16 runs was used; the design included an extra three replicate center runs; trials where all four factors are simultaneously held at their center value. Table 2 lists the four experimental factors and experimental levels, while the 16 experimental trial settings and results are shown in Table 3. The JMP^®^ Pro 15 statistical software was used to generate the design and perform the analyses. The analysis presented here focuses on the response titer after 48 h of fermentation time (see Table 3).

**Table 2:** Design factors and settings

Factor	Low	Middle	High
Temperature C	27	30	33
pH	6.5	6.8	7.1
DO %	30	35	40
Phosphate content (mM)	40.1	58.9	86.4

**Table 3:** Definitive screening design and experimental results

Conditions	Temperature (°C)	pH	DO (%)	Phosphate content (mM)	OD48 (600 nm)	DCW48 (g/L)	WCW48 (%)	Titer48 (g/L)
1	27	6.5	30	86.4	144	65.6	17.8	0.930
2	33	6.5	40	86.4	193	63.3	34.6	2.450
3	33	7.1	40	40.1	161	51.4	26.45	1.691
4	30	7.1	40	86.4	256	77	35.85	2.335
5	33	6.5	35	40.1	125.4	50.9	25.9	1.363
6	27	6.8	40	40.1	128	51.9	25.1	1.191
7	27	7.1	30	40.1	135.8	55	26.75	1.275
8	27	7.1	35	86.4	157.6	69.5	36.45	1.670
9	27	6.5	40	58.9	169.2	69.4	32.9	1.216
10	30	6.8	35	58.9	149.8	63.8	31.3	2.084
11	30	6.5	30	40.1	123.2	51.9	27.5	1.272
12	33	6.8	30	86.4	181	67.3	32.45	2.404
13	33	7.1	30	58.9	206	66.5	32.05	2.695
14	30	6.8	35	58.9	198	65.3	34.9	2.116
15	30	6.8	35	58.9	196	67.3	32.2	1.868
16	30	6.8	35	58.9	192	65.8	30.9	2.138

**Fig. 4:**
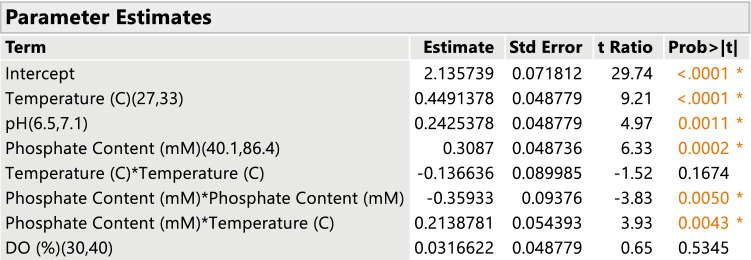
The fitted titer model

Given the goal of the experiment is to characterize and optimize performance, it is important to find an empirical model that best predicts fermenter performance in terms of titer. The model building strategy is based upon defining the full quadratic model for the four factors; there are 15 possible terms in the full model including an intercept. However, in actual practice, only a subset of these terms is likely to be important for the prediction of the response; statisticians refer to this as the effect sparsity principle [5]. The goal of the analysis to identify that subset of potential effects that results in the best prediction of titer. There are quite a number of approaches to model building (the process of finding the best subset) that can be used for DSDs and a full discussion is beyond our scope. A full discussion of model selection or model building techniques can be found in [19]. For these data, a new predictive modeling strategy developed by Gotwalt and Ramsey [6] was employed; please see the reference for full details. The method uses a combination of bootstrapping and model selection techniques to find those effects from a full quadratic model that are most important to accurate prediction of titer. The final model selected is displayed in Fig. 4 and consists of linear and quadratic effects of Salt feed and temperature, while DO % and pH are only linear effects.

**Fig. 5:**
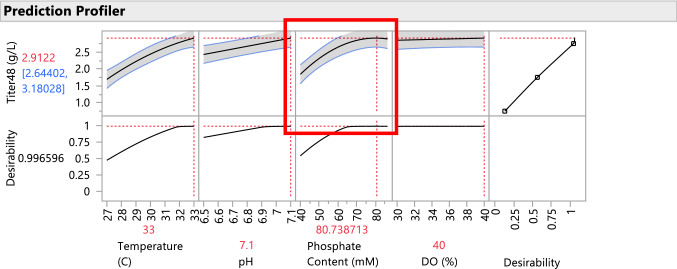
Prediction Profiler display for the fitted predictive model. Notice strong curvature in the relationship between titer and phosphate content

**Fig. 6:**
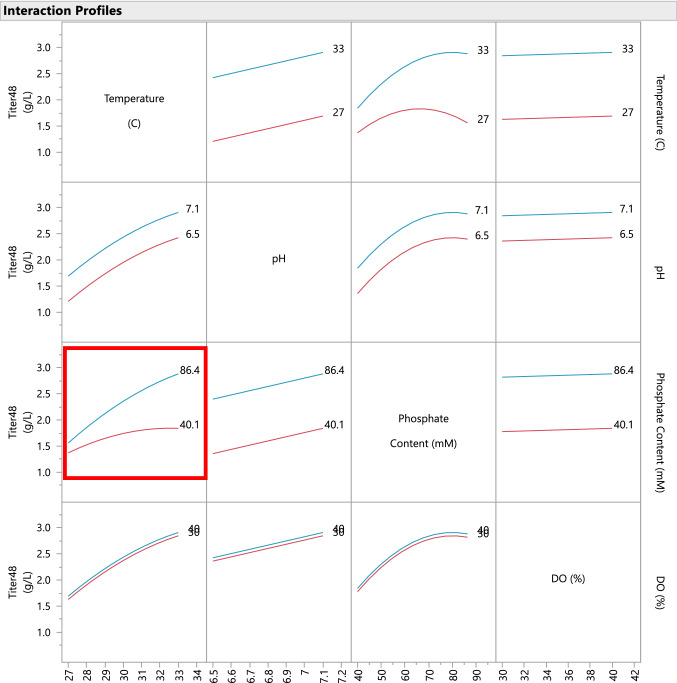
Interaction profiles for the fitted model. Notice in the lower left-hand corner is displayed the interaction plot for temperature (X-axis) and phosphate content. At low temperature, the effect of salt feed on titer is small; however, at high temperature, the effect is large

The fitted model can be visually displayed and evaluated in JMP using the Prediction Profiler, which is a dynamic, interactive visualization tool that allows the experimenter to physically interpret the behavior of the response as a function of the model. Figure 5 displays the Prediction Profiler for the titer model. The fitted model also includes an interaction between temperature and phosphate content, which indicates that the two factors do not act independently upon the titer response. Using an interaction plot, we can characterize the interactive behavior. Figure 6 displays a matrix of two-way interaction plots for the fitted model. From the interaction plot for temperature and phosphate content, it is apparent that phosphate content has a large positive effect on titer only at higher temperatures and the effect is small for low temperatures. To optimize titer, both of these variables must be simultaneously controlled.

**Fig. 7:**
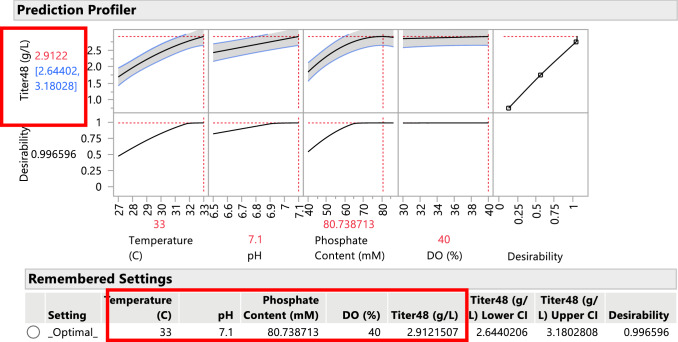
Prediction Profiler with optimized settings for highest average titer using desirability as the objective function. Notice that the highest titer = 2.885 (g/L) is achieve with all experimental factors at their high level (phosphate content of 86.4 mM)

Since the fitted model was selected as the best predictor of titer as a function of the design variables, the model can be subsequently used to optimize the fermentation process to achieve higher titer values on average. The optimization is performed using desirability functions, that is, the method searches for settings of the experimental factors, using the fitted models, such that optimum response levels are attained for all responses of interest. The Prediction Profiler in the JMP 15 software performs the desirability analysis and optimization. Figure 7 displays the optimized settings to achieve the highest titer values. The highest titer value achievable from the experimental results is on average 2.885 (g/L) and occurs when all four experimental factors are set to their highest levels.

**Fig. 8:**
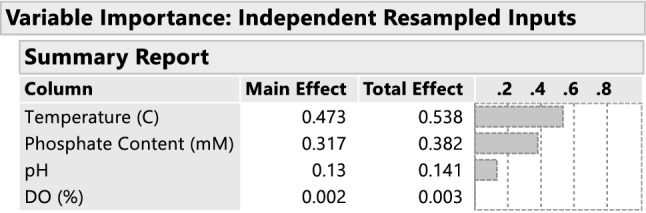
Variable Importance report. Notice that temperature and phosphate content explain almost all of the variation in titer, while DO (%) explains almost no variation. As a result, temperature and phosphate content are key input variables that must be tightly controlled in production

An important follow-on topic is the relative importance of the experimental inputs to the collective responses. The JMP Prediction Profiler incorporates a variable importance analysis, which is based upon functional decomposition methods from applied math; see Saltelli [21] or Sabol [26] combined with Monte Carlo simulations. The analysis allows the analyst to determine the relative contributions to variation in the response that is attributable to each experimental input in total, that is, including higher order terms and interactions. Figure 8 displays the results of the variable importance analysis. From the variable importance report, we see that temperature and phosphate content explain most variation in titer both as main effects and through their quadratic and interaction effects. Overall, DO (%) explains little variation in Titer over this experimental region. In production, it is important to tightly control both temperature and phosphate content given the sensitivity of titer to these two factors.

## Conclusions

Development of a process involving phosphate inducible promoter-based protein expression system has been presented. The goal is to establish the concentration range of phosphate to use for protein expression and biomass production beyond levels previously reported in the literature. The observations from this study follow the trend reported in the literature where an increase in the phosphate concentration tested in *phoA* process (10.8 mM − 18.0 mM) [27] and (0.43 mM − 17.0 mM) [8] improved biomass and titers. The results from this work is consistent with previous studies except that higher range of phosphate concentration (2.79 mM − 86.4 mM) were tested resulting in higher biomass and product titers. This study demonstrates that both biomass production and protein expression levels can be greatly improved when the initial concentration of phosphate in the batch is increased. The data from the scouting work were used for DoE where four factors, temperature, pH, DO content and phosphate content were simultaneously evaluated. Definite screening design was used to perform this analysis using JMP. The product titer was analyzed, and a model was developed to look at the impact of the process parameters on the product titer. The model showed that temperature and phosphate content were the major factors that impacted product titer. The approach used in this work can be applied by other workers developing therapeutic products using the *PhoA* system to improve cell biomass and product titers.

## Electronic supplementary material

Below is the link to the electronic supplementary material.

Supplementary material 1 (DOCX 14 kb)
