# Supplementary material for: Upstream development of Escherichia coli fermentation process with PhoA promoter using design of experiments (DoE)
Source: J Ind Microbiol Biotechnol. 2020 Aug 25;47(9):789–99. doi: 10.1007/s10295-020-02302-7 (PMC7658055; doi:10.1007/s10295-020-02302-7)
Supplement: Supplementary file 1 — Supplementary material 1 (DOCX 14 kb) [file 10295_2020_2302_MOESM1_ESM.docx]

**Table 1:** Mineral Salt Solution Composition used for fermentation. Each amount is the quantity (g) added per liter for each condition.

| Components | Concentration for 2.79 mM  (g/L) | Concentration for 27.1 mM  (g/L) | Concentration for 40.1 mM  (g/L) | Concentration for 58.9 mM  (g/L) | Concentration for 86.4 mM  (g/L) |
| --- | --- | --- | --- | --- | --- |
| FeCl_3_.6H_2_O | 1.8 | 1.8 | 1.8 | 1.8 | 1.8 |
| NaCitrate.2H_2_O | 5 | 5 | 5 | 5 | 5 |
| (NH_4_)_2_SO_4_ | 25 | 25 | 25 | 25 | 25 |
| K_2_HPO_4_ | 2.6 | 26.0 | 39.0 | 52.0 | 65.0 |
| NaH_2_PO_4_ | 1.3 | 13.0 | 19.5 | 26.0 | 32.5 |
| KH_2_PO_4_ | 1.5 | 15.0 | 22.5 | 30.0 | 37.5 |
| CuSO_4_ | 0.160 | 0.160 | 0.160 | 0.160 | 0.160 |
| ZnSO_4_.7H_2_O | 0.285 | 0.285 | 0.285 | 0.285 | 0.285 |
| CoCl_2_.6H_2_O | 0.257 | 0.257 | 0.257 | 0.257 | 0.257 |
| Na_2_MoO_4_ | 0.140 | 0.140 | 0.140 | 0.140 | 0.140 |
| H_3_BO_3_ | 0.040 | 0.040 | 0.040 | 0.040 | 0.040 |
| MnSO_4_.H_2_O | 0.112 | 0.112 | 0.112 | 0.112 | 0.112 |
